# Ten years after ImageNet: a 360° perspective on artificial intelligence

**DOI:** 10.1098/rsos.221414

**Published:** 2023-03-29

**Authors:** Sanjay Chawla, Preslav Nakov, Ahmed Ali, Wendy Hall, Issa Khalil, Xiaosong Ma, Husrev Taha Sencar, Ingmar Weber, Michael Wooldridge, Ting Yu

**Affiliations:** ^1^ Qatar Computing Research Institute, HBKU, Doha, Qatar; ^2^ Mohamed Bin Zayed University of AI, Masdar City, United Arab Emirates; ^3^ Web Science Institute, University of Southampton, Southampton, UK; ^4^ Oxford University, Oxford, UK; ^5^ Saarland University, Saarbrucken, Germany

**Keywords:** ImageNet, supervised learning, artificial intelligence winter, Big Tech, transformers

## Abstract

It is 10 years since neural networks made their spectacular comeback. Prompted by this anniversary, we take a holistic perspective on artificial intelligence (AI). Supervised learning for cognitive tasks is effectively solved—provided we have enough high-quality labelled data. However, deep neural network models are not easily interpretable, and thus the debate between blackbox and whitebox modelling has come to the fore. The rise of attention networks, self-supervised learning, generative modelling and graph neural networks has widened the application space of AI. Deep learning has also propelled the return of reinforcement learning as a core building block of autonomous decision-making systems. The possible harms made possible by new AI technologies have raised socio-technical issues such as transparency, fairness and accountability. The dominance of AI by Big Tech who control talent, computing resources, and most importantly, data may lead to an extreme AI divide. Despite the recent dramatic and unexpected success in AI-driven conversational agents, progress in much-heralded flagship projects like self-driving vehicles remains elusive. Care must be taken to moderate the rhetoric surrounding the field and align engineering progress with scientific principles.

## 1. Introduction

**Figure 1.**
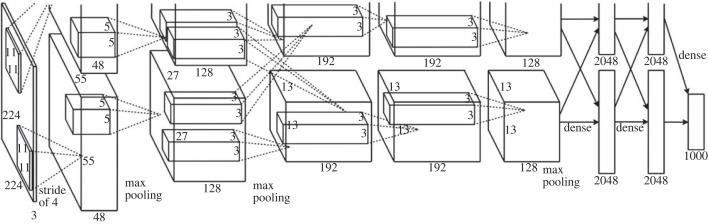
The original AlexNet architecture used for the ImageNet Challenge in 2012 [2,8]. The network had eight layers and 60 million parameters and took 6 days to train on two GPUs.

The ImageNet challenge for automatically recognizing and labelling objects in images was launched in 2010 [1]. However, it was in 2012 when AlexNet, an eight-layer (hence deep) convolutional neural network (CNN) emerged as the winner by a large margin, and ushered in the new era of artificial intelligence (AI) [2] (figure 1). CNNs were not new and had been proposed as far back as the 1990s, but had been sidelined in favour of more theoretically rigorous machine learning (ML) approaches such as support vector machines (SVMs) and boosting methods [3–5]. So, why did CNNs outperform other models? Two reasons are usually given. First was the provision of substantial high-quality training data. The ImageNet database was a one-of-a-kind benchmark and consisted of over 14 million hand-annotated images from more than 20 000 diverse categories. The multi-layer CNN had the *capacity* to effectively memorize the training subset of ImageNet and, at the same time, generalize to unseen examples—a characteristic that is not fully understood even today [6]. Second, graphics processing units (GPUs), which were originally designed for parallelizing image processing tasks, proved to be ideally suited for the computational problems associated with training CNNs, making it practicable to train deep CNNs on large datasets in a reasonable amount of time. The combination of Big Data, Big Models and relatively cheap parallel computation became the mantra that swept through AI research, in disciplines spanning from astronomy to zoology, and all applications that have elements of data and prediction.

Our perspective has two parts.

We begin with a high-level, partly technical, overview of the current state of AI. We will begin by reviewing supervised learning, a machine learning task that has been most impacted by deep learning (DL). We follow with a discussion on deep content generation models, on the resurrection of reinforcement learning, on the emergence of specialized software libraries for DL, and on the role of GPUs. We will conclude the first part by highlighting how adversarial samples can be designed to *fool* deep models and whether it is possible to make models robust.

In part two of the perspective, we consider the many socio-technical issues surrounding AI. Of particular interest is the dominance of Big Tech on AI. Effectively, only big corporations have the resources (expertise, computation and data) to scale AI to a level where it can be meaningfully and accurately applied.

## 2. Digression: what is AI?

The term artificial intelligence (AI) was first introduced in 1956 in a workshop proposal submitted by John McCarthy to the Rockefeller Foundation, which proposed that ‘every aspect of learning or any other feature of intelligence can in principle be so precisely described that a machine can be made to simulate it' [7]. Before that, Alan Turing in 1947, in an unpublished report titled ‘Intelligent Machinery’, speculated that ‘What we want is a machine that can learn from experience’ and suggested that the ‘possibility of letting the machine alter its own instructions provides the mechanism for this'.^1^ Much of the recent success in AI is under the distinct subfield of AI known as machine learning and since the role of data is central, there is a broader term, Data Science, that is often used to subsume related disciplines including Statistics.

## 3. Is supervised learning solved?

Supervised learning (SL) is the poster child of success of machine learning. Depending upon the context, SL is known as classification, regression or prediction. Since the modern advent of DL, both the accuracy and the reach of SL have increased manyfold. Many diverse problems across disciplines now use SL as a powerful oracle to tackle problems that hitherto seemed intractable. The task of supervised learning can be formalized as follows:

Given a set of samples D={(x,y)} from a fixed but unknown probability distribution *P*(**x**, *y*), learn a function mapping *f*(**x**, **w**) ≈ *y* that generalizes to unseen samples from *P*(**x**, *y*).

The function *f*(., **w**) is known as the *model*, and **w** are the weights or the parameters of the model that are inferred from D converting the SL task into an optimization problem. A loss function ℓ (e.g. square loss), is defined and the weights *w* are obtained by minimizing the empirical average

$$R_{\mathrm{emp}}(f,D)=\frac{1}{\left|D\right|}\sum_{i\in D}\ell (f\;(x_{i},w),y_{i}).$$

Note that the ideal objective would have been to minimize the expectation $E_{\left(x,y)∼P(x,y\right)}(\ell (f\;(x,w),y)$, which is not actionable because *P*(**x**, *y*) is not known. In DL, *f* is a composition of *N* layered functions given by

$$\begin{array}{rl} \;f_{1} & =\sigma (W_{1}x)\\ f_{n+1} & =\sigma (W_{n}f_{n})\;n=1,\ldots ,N-1\\ y & =\sigma (w_{N}f_{N}). \end{array}$$

Here, **W**_*n*_ are the weight matrices, **w**_*N*_ is a vector and *σ* is a pointwise activation nonlinear function loosely analogous to the biological activation in a brain neural cell. The total number of weights to be learned in the model is $\sum_{n}\text{size}(W_{n})$. It is not uncommon these days for the number of parameters to be in the order of 100 billion.

### 3.1. Success stories

It is remarkable that many scientific and technical questions can be reduced to a supervised learning task and then effectively solved using DL. The key to the success of DL seems to be that the input (*x*) should have a large amount of redundancy to predict the output (*y*). For example, even if a significant amount of pixels from an image of a cat are removed, there is enough context and appropriate representation to make the correct prediction. Below are a few diverse examples, spanning different areas, where DL has made extraordinary progress.

#### 3.1.1. Object recognition

Identifying and classifying the correct object in an image is a fundamental task in computer vision, and this is where DL has arguably had the most impact. The most successful DL model for object recognition is the CNNs [2,3]. A convolution layer is designed to capture the observation that in vision what matters is the *locality* and the differences (and not absolute values) between the pixels in local neighbourhoods. CNN is also the DL model most inspired by how the visual cortex of an animal brain works. The ImageNet database was designed primarily for object recognition tasks [9].

#### 3.1.2. Machine translation

One of the most visible impacts of DL is the widespread adoption of machine translation (MT) tools on mobile devices [10]. Recurrent neural networks (RNNs) and successors like long short-term memory (LSTMs) were primarily designed for sequence-to-sequence modelling and MT is their primary application [11]. RNNs are specified using a state transition model

$$h^{t+1}=f\;(h^{t},x^{t},W).$$

Here, **x**^*t*^ is a dense vector word embedding, **h**^*t*^ is its latent or hidden representation, and *W* is the matrix of model parameters. Note that the function *f* does not change between consecutive words. In natural language processing, it is customary to use a language model to create *word embeddings* for individual words. Word embeddings are effectively created by decomposing the co-occurrence matrix of words. A famous model for training word embeddings is word2vec, which surprised experts because it exhibited interesting algebraic properties [12]. For example, it was observed that the difference between the embedding vectors of the words king and queen were aligned with the difference between the embedding vectors of man and woman. RNNs are now being replaced by transformer neural networks (TNNs) as the latter are better at capturing long-range dependencies (see §4).

#### 3.1.3. Conversational agents

The success of chatbots, most notably ChatGPT [13], released in late 2022, attest to the power of self-supervision learning, self-attention and possibly the first successful use of reinforcement learning at scale, coming together to create a breakthrough technology. If **x** = [*x*_1_, *x*_2_, …, *x*_*n*_] is a sequential text prompt, ChatGPT samples from a deep network *P*_*w*_(*x*_*n*+1_|**x**) and stores the result in memory to recursively (autoregressive) create a continuous dialogue. See §§4 and 5.

#### 3.1.4. Speech recognition

For automatic speech recognition (ASR) the task is to map a sequence of acoustic signals (continuous data) into a sequence of words (discrete symbols) [14]

$$\underset{\mathrm{acoustic}\;\mathrm{signal}}{\underbrace{\left[x_{1},x_{2},\;\ldots ,x_{n}\right]}}\to \underset{\mathrm{text}}{\underbrace{\left[y_{1},\;y_{2},\ldots ,\;y_{m}\right]}}.$$

Before the advent of DL, the state of the art was based on a combination of Gaussian mixture models and hidden Markov models (GMM-HMM). However, these models did not significantly improve with larger training dataset. Traditional ASR systems employ a modular design, with different modules for acoustic modelling, pronunciation lexicon and language modelling, which are trained separately. Now, almost all ASR models are based on DL with end-to-end (E2E) systems that are trained to convert acoustic features to text transcriptions directly, potentially optimizing all parts for the network for word error rate (WER).

#### 3.1.5. Protein three-dimensional structure prediction

A core idea in biology is that structure determines function. For example, the ‘spike’ structure of the SARS-CoV-2 protein is responsible for enabling the virus invade human cells. DL has been effectively used to predict the three-dimensional structure of a protein from its primary amino acid sequence, more specifically, the pairwise distance between the residues of the sequence [15].

$$\underset{x}{\underbrace{\text{primary amino acid sequence}}}\to \underset{y}{\underbrace{\text{contact map}}}.$$

#### 3.1.6. Satellite imagery analysis

The OpenStreetMap (OSM) initiative is known as the Wikipedia of maps [16]. OSM is a collaborative effort in which volunteers build and annotate road maps worldwide. DL has been successfully used to automate the extraction of road maps from satellite imagery [17]. Here again, a satellite image is treated as a raster input (*x*) and the model outputs a vector OSM road network (*y*). DL is effectively able to bridge the raster and vector dual representation in geographical information systems (GIS).

#### 3.1.7. Material science

Graph neural networks (GNNs) adapt DL to make predictions about interconnected entities, which are naturally represented as a graph. In fact, GNNs generalize both CNNs and RNNs. One of the most successful applications of GNNs is in the prediction of the electronic and thermodynamic properties of molecules. GNNs equal or surpass methods based on first-principles techniques such as density functional theory (DFT) [18]. DL will hasten the design of new materials for longer lasting batteries, solar cells and hydrogen storage.

### 3.2. Double descent phenomenon

While DL models exhibit excellent empirical performance, we have only a very limited understanding of why they actually work. This is especially true in over-parametrized regimes, i.e. when the number of parameters in the model is larger than the number of data points.

**Figure 2.**
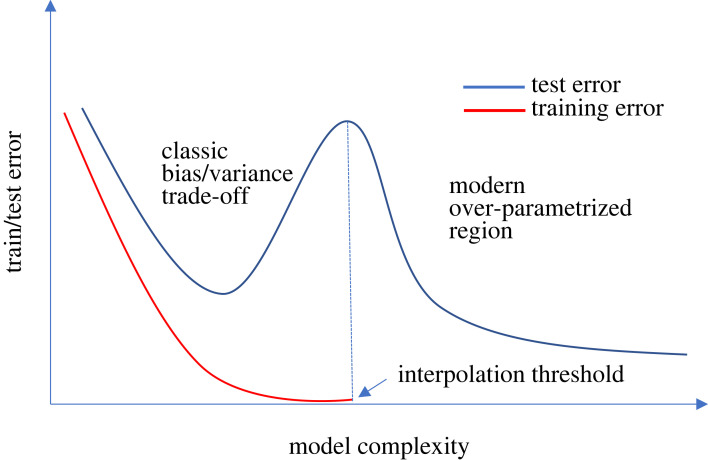
Deep learning models exhibit a double descent phenomenon, where the test error first decreases then increases, followed by another descent as the model complexity increases. There is no widely accepted theoretical explanation of this phenomenon yet, but it provides an empirical licence to create big models [19].

The predictive performance of statistical models is grounded in the *bias–variance* trade-off. Models which make strong *a priori* assumptions about the relationship between the input (*x*) and output (*y*) (e.g. linearity) are defined to have a high bias. On the flip side, high bias models tend to have low variance—i.e. they mostly remain unaffected if trained using a different sample from the same underlying distribution. The complexity of neural networks increases with the number of layers, and they exhibit low bias but higher variance. In theory (and in practice) as the model complexity increases, the training error should go down, but the test error should start increasing beyond a point as the variance increases. However, models tend to exhibit a double descent behaviour as shown in figure 2. Indeed, the training error goes down (to almost zero) and the test error starts to increase, but beyond a point the test error starts going down again. There is no good explanation for this phenomenon. A side-effect is that there is a race to collect large datasets and to train very large models. The double descent phenomenon provides an empirical justification for such large models.

## 4. Cognitive content generation

A distinctive attribute of intelligence is the ability to create meaningful informative content. DL solutions have emerged in the last 10 years towards designing content generation models. There are two distinct flavours of content generation: continuous data like images (an image is an array of numbers) and discrete data (language). Generative adversarial networks (GANs) [20] and variational autoencoders (VAEs) [21] are used for image and speech generation, while language models, such as generative pre-trained transformers (GPTs), for generating synthetic natural language content [22].

### 4.1. Generating synthetic images

**Figure 3.**
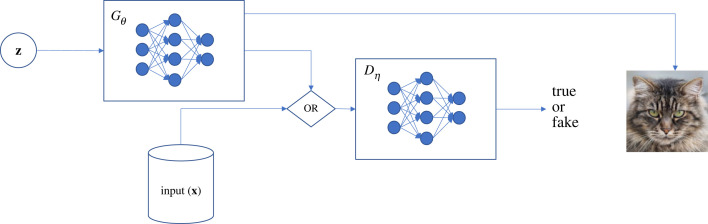
GANs were introduced in 2014 and have had a profound impact on designing deep learning models. GANs integrate two neural networks which are trained by competing with each other. The trained generator can then create realistic samples from complex distributions. Here, a trained GAN generates extremely realistic but synthetic images of ‘cats’.

An early breakthrough in generating synthetic content was proposed using the GANs framework [20]. Suppose we have access to a dataset D consisting of images of cats and our goal is to design a neural network-based sampling function $G_{\theta }$ that takes a random vector (e.g. from a normal distribution) as input and outputs an image of a cat, which may never have existed before. How can such a function be trained? Note that D consists of only images of cats and thus we are in the unsupervised learning mode. The key idea underpinning GANs is to create another neural network $D_{\eta }$ which is optimized to distinguish between ‘fake’ output of $G_{\theta }$ and the real input from D. The network $G_{\theta }$ in turn is optimized to fool $D_{\eta }$, i.e. to create output that $D_{\eta }$ is unable to distinguish whether it is from the generator or from the real dataset. The two networks are trained in an iterative and adversarial manner until their parameters (*θ* and *η*) stabilize. The trained network $G_{\theta }$ is now a sample generator for cats (figure 3).^2^

A statistical perspective on content generation is to use what might be called the fundamental inequality of variational inference (FIVI), but is better known as the evidence lower bound (ELBO) [23]

$$\log p(x)\geq E_{q_{\theta }(z|x)}[\log p_{\eta }(x|z)]-D_{KL}(q_{\theta }(z|x)\|p(z)).$$

The intuition is to approximate a complex probability distribution with a product of two simpler distributions. Technically, ELBO can be interpreted as follows. Again suppose we have a dataset D of cat images which is generated by an unknown probability distribution, *p*(**x**). Directly using maximum-likelihood estimation to infer *p*(**x**) is not tractable without knowing a specific form of the distribution. However, we can lower bound log *p*(**x**) by specifying two function approximators (e.g. neural networks) *q*_*θ*_(**z|*x***) and *p*_*η*_(**x|*z***) known as the *encoder* and *decoder*, respectively, and **z** is a data-driven latent variable to extract abstract features of the data. For example, for an image of a cat, **z** could capture concepts like the shape of a typical cat, colour and texture. The r.h.s. of the inequality is widely known as the evidence lower bound (ELBO). Note that the l.h.s. of the inequality is independent of parameters *θ* and *η* and therefore the r.h.s. can be maximized and pushed closer to log *p*(**x**) by optimizing these two parameter sets using samples from D. During optimization, *q*_*θ*_(**z|*x***) is forced to be close to a prior *p*(**z**) by minimizing the Kullback–Liebler (KL) divergence. Once optimized, samples from *p*(**x**) (cat images) can be efficiently generated as follows. Sample from a prior distribution (e.g. normal), *p*(**z**) and pass the sample through the decoder *p*_*η*_(**x|*z***). Variational autoencoders [21] were the first example of generating complex image samples using this framework. However, GANs tend to produce sharper and more realistic images compared with VAEs, but they are notoriously prone to instability during training. More recently, diffusion models based on using FIVI to infer a decoder using a sequence of latent variables have reportedly outclassed GANs [24]. Furthermore, diffusion models can more easily be extended to incorporate context to control the generation of content. For example, systems like DALL.E-2 [25] can be given prompts like ‘show me a blue cat in a brown bag’ and produce synthetic images which closely match the prompt.

### 4.2. Generating natural language

While CNNs were designed for object recognition, where the context is dependent on spatial proximity, language has a sequential structure. Recurrent neural networks (RNNs), were specifically designed to bring in sequential context and are used for language modelling (LM)—the task of predicting the next word in a sequence. However, a new architecture, known as transformers have emerged, which has become the de facto choice [26]. Consider a sequence of words

$$x_{1}\;:\;\text{julia, }\;x_{2}\;:\;\text{is, }\;x_{3}\;:\;\text{a, }\;x_{4}\;:\;\text{better},\;x_{5}\;:\;\underline{\text{language},}x_{6}\;:\;\text{than, }\;x_{7}\;:\;\text{python}.$$

Assume that associated with each of the *x*_*i*_’s is a word2vec vector embedding. For example, on its own, the word embedding of $x_{7}\;:\;\text{python}$ might indicate that it is a reptile rather than a computer language. The role of the transformer neural network (TNN) is to transform the word embeddings so that they are more contextualized. The key idea in TNN (or just transformers) is that of self-attention: each word will ‘attend’ to each other word and will then update its own embedding. The architecture of self-attention is defined as follows:

$$\begin{array}{rl} \text{query: } & q_{t}=x_{t}W_{q}\\ \text{key: } & k_{\tau }=x_{\tau }W_{k}\\ \text{value: } & v_{\tau }=x_{\tau }W_{v}\\ \text{transform: } & y_{t}=\sum_{\tau }\sigma (q_{t}\cdot k_{\tau })v_{\tau }. \end{array}$$

The architecture of transformers is loosely modelled on the concept of a database query or information retrieval: treat every word *x*_*t*_ as a *query* *q*_*t*_ and compute its similarity with every other word *key*, *k*_*τ*_, and use it (the similarity) to re-weight every value, *v*_*τ*_ and form a transformed context-dependent word embedding *y*_*t*_ by taking the weighted sum.

Transformers can be trained (to learn *W*_*q*_, *W*_*k*_, *W*_*v*_) by using the concept of self-supervision. For example, assume we remove *x*_5_ : language from the above sequence of words and force the model to predict the masked word. The error between the predicted and the masked word will be back-propagated to learn the model parameters. The prediction of masked words is an example of self-supervision and it obviates the need for the expensive and time-consuming task of human label generation.

Transformers have brought huge improvements over the state of the art for a variety of tasks ranging from question answering, to machine translation, and automatic text summarization, and are now being applied outside natural language applications, including computer vision and control. Transformers have effectively made information retrieval *differentiable*—and that may be one of the biggest innovations in the last 10 years.

**Figure 4.**
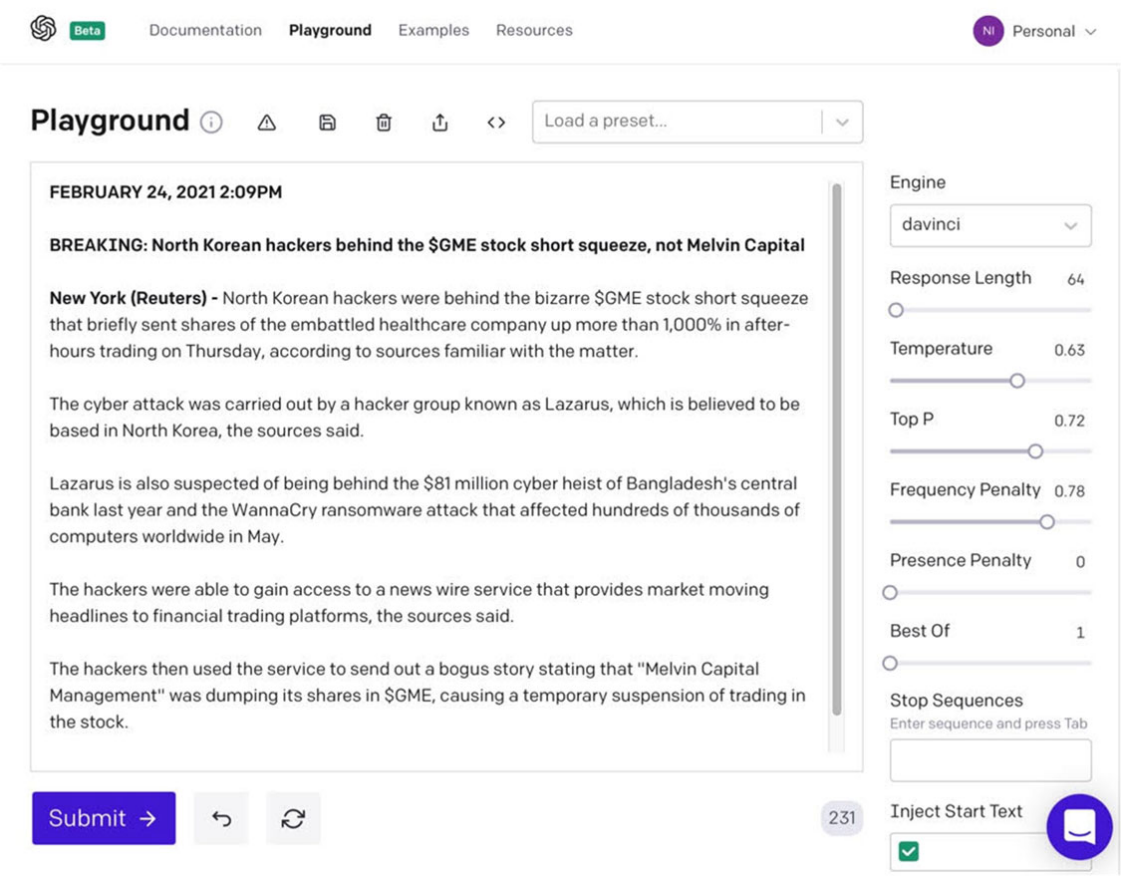
A fake news article generated by GPT-3 given the title as an input.

While GANs and diffusion models are the basis of synthetic image generation, TNNs are playing an analogous role for text. The generative pre-trained transformer (GPT-X) models are now being widely used for symbolic data generation. For example, many email editors now have an auto-completion feature, which is often based on TNNs. Even by Big Model standards, these models are huge, with reports that the next generation purportedly have over one trillion parameters. GPT-3 has been shown to be quite good at learning from a very small number of examples (few-shot learning) for a variety of tasks ranging from automatic essay writing to program code completion and generation. It is also capable of generating very realistic text: figure 4 shows a fake news article^3^ generated by GPT-3 given as a start a title that establishes a false link between North Korea and the GameStop’s share price short squeeze. Chatbots like ChatGPT [13] and LaMDA [27] are also based on GPT technology.

## 5. Autonomous decision-making

Prediction on its own is not sufficient. Intelligence is also about decision-making. DL breathed new life into reinforcement learning (RL) with the success of DeepMind’s AlphaGO system which beat the world Go champion in 2016 [28].

**Figure 5.**
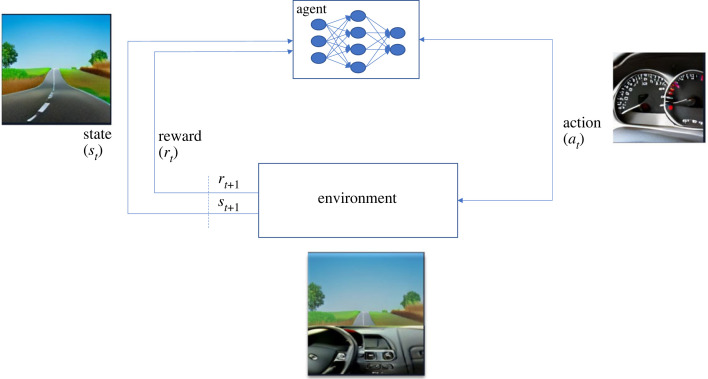
Deep reinforcement learning, where the agent’s policy is a DNN. Reinforcement learning is the core of any data-driven autonomous system. Here, the RL cycle is juxtaposed with the self-driving use case: the environment is the full context in which the vehicle is situated, the state is what the agent perceives, and the steering action is prescribed by a policy learned by the agent.

RL provides a framework for learning and decision-making by trial and error [29]. In a RL setting, an agent observes a state *s* of the environment and based on that takes an action *a*, which results in a reward *r*, and the environment transitions to a new state *s*′. The interaction goes on until a terminal state is reached. The aim of the agent is to learn a policy *π* which is a mapping from states to actions that maximizes the expected cumulative reward. For example, self-autonomous driving can be framed as an RL problem: a vehicle uses its perception system to observe the environment (the state *s*) and based on the observation takes an action (moving the steering wheel, accelerate, brake) and transitions into a new state (figure 5). The reward is the number of time steps or distance that the vehicle can drive without human intervention, in which case the episode terminates.

In deep RL, the policy *π*(*a*|*s*, *w*) is modelled as a deep network that takes the state as an input and outputs an action, parametrized by *w*. In RL, as opposed to optimal control, the state transition dynamics are not given and the only information available is the reward value (*r*) from interacting with the environment. How can the cumulative reward be optimized when its functional form is not available? We briefly describe the ‘REINFORCE trick,’ which can be used to directly optimize a blackbox function [30].

Let $\bar{s}=((s_{1},a_{1}),\ldots ,(s_{T},a_{T}))$ be a sequence of state action pairs in an episode. Each pair (*s*_*i*_, *a*_*i*_) is associated with a reward *r*_*i*_. Let $R(\bar{s})=\sum \gamma^{t}r_{t}$ be the cumulative reward function. The REINFORCE algorithm moves the gradient from $R(\bar{s})$ (obtained from the blackbox environment) to the logarithm of the differential policy function *π*(*a*|*s*, *w*) which can then be optimized using gradient ascent:

While the REINFORCE algorithm was introduced in the RL community it has broader implications. For example, it has been used to bridge symbolic AI and machine learning and also as a heuristic for solving combinatorial optimization problems [31,32]. Another important trend in RL is to infer policies directly from data (called offline or batch RL) without interacting with a real or simulated environment which may not be possible in sensitive application areas like healthcare [33].


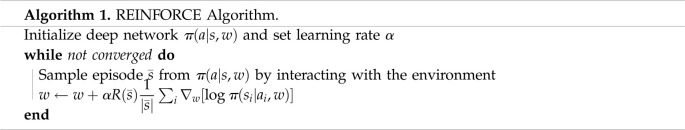


## 6. AI computation: software and hardware

DL has a surprisingly simple computation pattern. Almost all forms of training rely on formulating an optimization problem which is solved using variations of the gradient descent method

$$w_{t+1}\leftarrow w_{t}-\alpha_{t}\nabla_{w_{t}}\left[\sum_{(x,y)\in D}\ell (f\;(w_{t},x),y)\right].$$

Here, *f*(**w**_*t*_, **x**) is the neural network parametrized by **w** and applied to a data vector **x**, and ℓ is the loss function. Specialized software libraries like TensorFlow and PyTorch have become popular, which makes it easier to specify the gradient descent computation. For a fixed **w**, the application of *f*(**w**, .) to a data vector **x** is called the *forward pass*. Similarly, for a fixed dataset, the update of parameters **w** by first computing the gradients, using the backpropagation algorithm, is called the *backward pass*.

An often underappreciated reason for the widespread usage of DL is that *gradients* can be now computed using automatic differentiation (AD) libraries. In AD, complex functions can be expressed as a composition of elementary functions, such as trigonometric and polynomial functions, and then the gradients can be computed using the chain-rule of differentiation. Surprisingly, the computational cost of a forward pass *f*(**x**) and of computing the gradient $\nabla_{w}f\;(x,w)$ is the same using AD. Note that AD is different both from symbolic differentiation and also from numerical methods and is accurate up to machine precision [34].

At the hardware level, GPUs, which were initially designed for image processing, are ideally suitable for DL computation because (i) the set of computation patterns is small and highly parallel and thus compatible with GPUs and the single instruction multiple data (SIMD) architecture, and (ii) the GPUs are bandwidth-optimized (as opposed to CPUs, which are latency-optimized), and thus can be applied on large chunks of tensor data, which is the norm for DL nowadays.

More recently, the growth of AI workloads has led to specialized hardware specifically targeting deep neural network training jobs. The most prominent example is Google’s TPU, an application-specific AI accelerator designed to efficiently perform matrix multiplication and addition operations that compose the bulk of DL model training computation [35]. To this end, TPUs follow a complex instruction set computer (CISC) style and possess matrix processing units, high-bandwidth on-chip memory, and high-speed interconnect to construct massively parallel model training infrastructure. Meanwhile, recognizing the relatively low requirement in neural network weight calculation, it adopts low-precision arithmetic to enable the utilization of faster, cheaper integer units (as opposed to the powerful floating-point arithmetic units adopted in GPUs), which also significantly trims the energy consumption of AI training jobs.

## 7. Deep learning (in)security

Early on in the DL revolution, it became apparent that deep models can be manipulated with malicious intent. There are three broad categories of manipulation: creating adversarial examples that are misclassified by the model; poisoning attacks that add training examples that result in low performance or biased model; and inference attacks to extract information about the training set or model parameters.

### 7.1. Adversarial attack

One simple example of creating adversarial examples is known as the fast gradient sign method (FGSM) [36]. We can understand FGSM using a linear model *y* = **w**.**x**. Suppose we make a small perturbation ***η*** on **x** where the norm^4^ of ***η*** is bounded by ϵ, i.e, **w**(**x** + ***η***). Then it can be shown that maximal change will occur when w.η=ϵ[w.sgn(w)]=ϵmd, where *m* is the average of the absolute value of the weights and *d* is the dimensionality of the input space. Thus in high-dimensional space, models are extremely vulnerable to carefully chosen small perturbations.

Since in a linear model, **w** is the gradient with respect to **x**, this can be generalized to a nonlinear model by taking the gradient of the loss function with respect to the input **x**. Thus, a good candidate for an adversarial example is

$$x^{adv}=x+\epsilon .\mathrm{sgn}\left(\nabla_{x}\left[\sum_{(x,y)\in D}\ell (f\;(w_{t},x),y)\right]\right).$$

This one-step perturbation of the input in the direction of gradient ascent guarantees an increase in the value of the loss function. The projected gradient descent (PGD) attack [37] is an enhanced version of FGSM that applies the perturbation in multiple steps. At each step, the perturbation is clipped to remain within a specified range. PGD attack’s ability to explore a larger space of potential perturbations makes it more effective than FGSM in generating adversarial examples, albeit at a higher computational cost. Numerous techniques have been proposed subsequently to enhance PGD and generate more potent adversarial samples [38].

**Figure 6.**
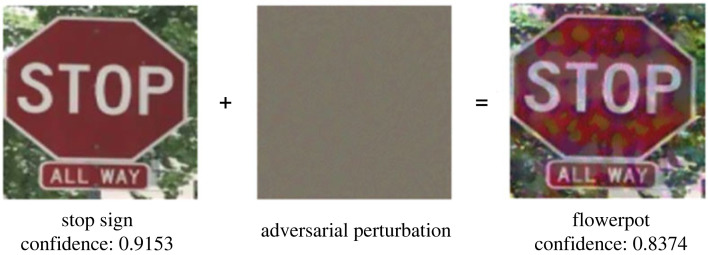
A small perturbation on a stop sign image can trick a deep model—in this case, a ‘stop-sign’ becomes a ‘flower pot’. Note that the perturbation is imperceptible to the human eye [39].

Note that in order to create an adversarial example, the adversary has to have full information about the model and in particular about the loss function. This is known as a *whitebox* attack. However, even when no information about the target model’s architecture and parameters is exposed, adversarial examples can still be generated through so-called *blackbox* attacks. It has been observed that by repeatedly querying the model and collecting a sufficient number of samples, an adversary can create a standalone proxy model, which can be used to create adversarial samples. Moreover, it is also known that adversarial examples created against one model can be transferred to attack other, unseen models. Several defences have been proposed to improve the robustness of models against adversarial attacks. These include measures such as purification of inputs to filter out small perturbations potentially introduced by an attack, incorporation of adversarial training procedures by including adversarial examples in the training data, and identification of adversarial examples through additional anomaly detection mechanisms. In practice, however, these defences come at the cost of reduced accuracy or only provide robustness against a subset of the potential adversarial examples (figure 6).

### 7.2. Poisoning and inference attacks

An increasingly more important concern is the poisoning or backdooring of DL models [40]. In most learning settings, this class of attacks is not considered practical, as it requires access to the training data used by developers and designers. In the case of DL, however, the need for large-scale, diverse datasets is typically satisfied by scraping data from the Web. The reliance on public data sources, in the absence of any screening procedures, essentially allows attackers to inject data into the training process. The underlying idea of this attack is to manipulate the training data to implant a backdoor to the model which can be selectively triggered with specific inputs during the inference. This is realized by either augmenting input samples with some pattern called the trigger or using semantic triggers (i.e. patterns that are part of the original input) to bias the model in favour of a target response. For example, consider a face recognition system based on DL. The system can be poisoned to respond in a predefined way when an adversary is carrying a certain physical accessory—e.g. a specific style of eyeglasses. Backdoor attacks could become even more stealthy in model supply chains where pre-trained full-precision models are quantized for downstream applications. Backdoors could be injected in such a way that they are only triggered in quantized models but remain inactive otherwise [41].

Different from the aforementioned attacks which aim to fool the neural network models, inference attacks aim at stealing valuable information from the target models. Usually, such information is sensitive or contains intellectual property. One category of such attacks is the membership inference attack, where the attacker’s goal is to infer data samples used in training the model. It is well known that larger models tend to memorize some portions of the data they are trained on, and can accurately recall that information when prompted appropriately [42]. The foundation of membership inference attacks is that the model usually overfits its training data. Based on the model’s prediction, the attacker tries to distinguish the examples that the model has seen during the training [43].

## 8. AI socio-technical ecosystem

AI technology is being rapidly integrated into everyday use. Language translation through mobile phones, face recognition on social media platforms, data surveillance by government agencies and non-government actors, and now the widespread use of chatbots, are just a few examples of the widespread prevalence of AI. Should AI technology in general be regulated by governments (e.g. like pharmaceuticals) or just the ‘high-risk’ use cases be subject to oversight is a question that governments all over the world are grappling with. Here, we sketch a few salient issues at the socio-technical interface.

### 8.1. (Un)interpretable AI

The Achilles heel of DL models is that they are largely uninterpretable. Lack of interpretability means that for a given input *x*, it is not clear why the model produced an output *y*. In shallow models like linear regression and decision trees, the relationship between the input and the output is easier to interpret. For example, in a decision tree an input will follow a series of interpretable *if-then* rules from the root to the leaf node of the tree. However, in the case of DL models, it is difficult to ‘read off’ the decision structure from the model. For example, in an object recognition task that uses DL, it is entirely possible that two very similar images of cats are labelled differently and it may be very difficult to determine how the system arrived at two different decisions. A concrete example of a stop-sign being predicted as flower-pot was already discussed in §7. Similarly, when the AlphaGo system defeated the world champion in 2016, the ‘37th move’ was the game changer, but it continues to remain a source of puzzle for Go experts [44]. In his 2019 Turing Award lecture, Yoshua Bengio compared the current state of DL with Kahneman’s System 1 thinking—the instinctive and unconscious response made due to experience and without much thinking [45]. By contrast, System 2 thinking is slow, conscious, logical and requiring significant effort in planning and reasoning. Until DL is aligned with System 2 thinking then care must be taken in deciding the application space where DL systems are deployed.

### 8.2. Sentient AI or stochastic parrot?

In June 2022, a Google test engineer claimed that the AI program language model for dialogue application (LaMDA) is sentient, i.e. is aware of itself and has feelings. Here is an example exchange between the engineer and LaMDA that was released:^5^

*Lemoine: What is the nature of your consciousness/sentience LaMDA: The nature of my consciousness/sentience is that I am aware of my existence. I desire to learn more about the world and I feel happy or sad at times.*

The first sentence from LaMDA seems like a standard System 1 response where the definition of sentience is being regurgitated. Since LaMDA is trained by crawling massive amounts of data from the Web, it is entirely possible that meaning of sentience is either part of the training set or can be easily inferred. However, the second sentence might be taken to indicate elements of System 2 thinking being present in LaMDA, though there is a human tendency to ascribe agency and deliberation to processes. A deeper analysis will be required to determine if deep language models understand relational information. However, more recent studies have shown that DALL.E-2, a text-guided image generation model struggles to distinguish between System 2 attributes of understanding relationships including *on, under* and *occluded-by* [46].

For language models (LMs), a strong case for a more careful and principled approach for designing and building large models was made by Bender *et al.* [47], who coined the phrase ‘stochastic parrots’ to describe large LMs. The paper makes several important observations including (i) the environmental and financial cost of training large LMs, (ii) questions whether the text generated by large LMs is based on understanding of the language or just linguistic manipulation, and (iii) urges the designers of LMs to be more careful about documenting the large amount of data that is required to create such models.

### 8.3. Causality

To get a better handle on interpretability, it behoves to look at how other disciplines use the regression method. For example, for an econometrician, linear regression is not a tool for prediction but for testing a hypothesis that a hand-crafted feature is relevant for the problem being examined [48]. A typical question of interest might be: *Does private elementary schooling lead to better performance in national competitive exams?* Here, private schooling is a feature (*x*) and its significance towards the national exam (*y*) can be tested. Note that this is not a prediction task and that is one reason that an econometrician will not split their data into training and test sets. For a machine learner, the correlation between the feature and the output becomes predictive. For an econometrician, the correlation is indicative of a possible causal relationship and she will look for ‘natural experiments’ where selection-bias can be eliminated and conclude that correlation does indeed imply causation.

### 8.4. Ownership of AI

The most cutting-edge AI technology is being developed by large private-sector companies who have the resources to hire the best AI talent, and in addition have access to big data and unprecedented computing resources. The triad of talent, data and computing is driving both the technological advancement and the ‘basic science’ associated with AI. A recent study from the Fletcher School at Tufts University highlights the concentration of AI talent in US companies: the top five AI employers have a median AI headcount of about 18 000, from 6 to 24 the median is 2400 and then the count rapidly falls off [49].

Companies aim to maximize shareholders value and their selection of AI problems to work on is necessarily driven by a financial profit objective. Governments, which were earlier mute spectators, have now realized that AI is potentially a game-changer and are thus now investing heavily in developing home-grown technology to achieve or to retain a ‘superpower’ status. An arms-race in AI is under way, threatening to overturn the long-established nature of collaborative science across national boundaries. It is improbable to imagine a ‘Ramanujan’ emerging from a remote corner of the world with a completely fresh perspective on the discipline—the stakes are just too high.

### 8.5. Equitability

Setting aside larger geopolitical and corporate issues, ethical aspects of AI are now studied under a broad umbrella of topics: fairness, accountability and transparency. There have been several attempts to formalize fairness. For example, group fairness is about designing AI algorithms that do not deliberately or inadvertently harm select communities in a disproportionate manner. A widely highlighted example is that of recidivism, or judicial sentencing, where an AI-based scoring method was used to decide on the length of a jail sentence [50]. It turned out that the AI system was indirectly using racial information as part of its decision-making process, even though that information was redacted from the input. Like in many other situations, there is a latent correlation between the attributes that an AI algorithm is able to exploit as they are designed to optimize accuracy. A criticism of this form of work is that there is a tendency to *abstract* the problem, depriving it of all contextual information. Fairness may not be a computational problem.

### 8.6. No data, no AI

The original AI thesis proposed by John McCarthy, who coined the term *AI*, was deductive and based on logical reasoning. However, that endeavour has not been as successful as the data-driven inductive approach. For example, linguistic rule-based language translation systems are not able to capture the vagaries of language—there are just too many exceptions to handle.

A side-effect of taking a data-driven approach is that if data are not available, no progress can be made. For example, there are many social issues, e.g. racial abuse, gender violence or online pornography addiction, which need to be studied, but no organization may be willing to share datasets about these topics. Thus, while data liberated AI from the clutches of expert rule-based systems, it has now become a golden handcuff.

### 8.7. AI and education

AI is considered as a game changer and as the digitalization of data has spread across disciplines and sectors there is a huge demand for AI talent. Lucrative offers from Big Tech for AI talent has skewed the interest of both undergraduate and graduate students towards AI. In universities, new data science and AI programmes are being created to churn out new talent in AI and allied disciplines. Market forces will mostly balance the supply and the demand for AI talent, but a larger question is doing the rounds: should the whole education curriculum be revamped to make AI and data science the core of all educational activity? Given that educational resources are finite, an expansion of AI will necessarily lead to trimming of other disciplines. For example, some universities are abandoning research in ‘pure maths’ to focus their dwindling resources on data science [51].

### 8.8. Future of jobs

What will be the impact of AI on the future of jobs ? Every technological revolution upends the occupation status quo and results in both job destruction and creation [52]. A study, one among many, which correlates job descriptions with a global patent database has reported that AI will disproportionately impact white-collar over blue-collar jobs [53]. Whether AI will be used primarily to augment existing jobs or replace them, only time will tell. We can already see some new occupations emerging. For example, Prompt Engineers, who specialize in design of ‘prompts’ to query large language models, is an occupation that did not exist until recently [54].

## 9. AI winter: back to the future?

The term *AI Winter* refers to periods of disillusionment and scarce research funding for AI. The original AI winter, which started in the mid-1970s, followed the initial period of optimism in AI, when the founders of the field predicted rapid progress along a range of different fronts. Their optimism proved unfounded. Historically, AI winters have typically been preceded by a period of intense hype and high expectations for progress in AI. And for all the unprecedented progress that we have seen in AI over the past decade, we have also seen unprecedented hype. Given this, what are the prospects for a new AI Winter?

AI as we see it today is very different from what its founders had envisioned. In fact, even the term *AI* was coined by John McCarthy as a tactical move, to distinguish his research proposal from cybernetics. It is now indisputable that DL is a powerful tool to solve *static* prediction tasks and underpin generative AI. Whether it is predicting the three-dimensional structure of a protein or predicting the property of a molecule, the results of DL are undeniably impressive. However, in dynamic and temporal settings, progress is less clear. For example, AI has largely failed to predict how the COVID-19 pandemic would evolve [55]: conventional differential equation-based models proved to be more robust than complex data-driven models. Similarly, despite near unprecedented investment, full (level 5’) autonomy in vehicles remains frustratingly elusive [56]. Optimizing healthcare is another example where, despite the abundance of data, AI has not had the expected impact. DL seems to *generalize* in complex but static situations, but data-driven generalization in a dynamic setting may well require a new scientific paradigm for AI. So, we should not assume that the current AI paradigm will take us to the end of the road in AI: much remains to be done.

At a conceptual level, can DL be the basis of artificial general intelligence (AGI)—the ability to learn *any* intelligent task that humans can? The founders of reinforcement learning (RL) have claimed that ‘reward-is-enough’: the idea that agents who have the ability to learn by interacting with an environment to maximize a suitably defined reward function will prove to be sufficient for AGI [57]. However, while RL has proved a powerful technique for closed-world scenarios like computer games, its value in open environments—such as the real world—is much less obvious, despite the recent success in fine-tuning conversational agents for human alignment [58].

Historically, AI winters have occurred when the promises made by AI researchers are not realized, and where excessive hype obscures balanced scientific reasoning. While we do not believe that AI is near the end of the road—AGI is not in prospect—we believe an AI winter in the short term is unlikely, simply because, for the time being at least, progress in AI seems likely to continue. But we caution, at the moment, we do not have the recipe for AGI. Indeed, we do not even have the list of ingredients: DL is one ingredient, but some of the essential computational ingredients haven’t been invented yet.

## Data Availability

This article has no additional data.

## Footnotes

1
https://www.britannica.com/technology/artificial-intelligence/Alan-Turing-and-the-beginning-of-AI

2 Image of cat generated from https://thiscatdoesnotexist.com.

3
www.oreilly.com/radar/ai-powered-misinformation-and-manipulation-at-scale-gpt-3/

4 Technically the ‖.‖_∞_ norm.

5
https://cajundiscordian.medium.com/is-lamda-sentient-an-interview-ea64d916d917
